# Challenges in the application of the Tshivenda scientific register for physical sciences classrooms

**DOI:** 10.12688/f1000research.131616.1

**Published:** 2023-06-16

**Authors:** Ndivhuwo P Netshivhumbe, Awelani V. Mudau

**Affiliations:** 1 science and technology education, university of south africa, pretoria, gauteng, South Africa

**Keywords:** Classroom practice, Tshivenda Scientific register; Physical Sciences; Challenges; Teachers; Learners

## Abstract

**Background:** This paper investigated some of the challenges in the application of Tshivenda scientific register (TSR) during classroom practices of some physical sciences teachers in some of the public secondary schools in the Vhembe West District, South Africa.

**Methods:** It was an interpretative qualitative case study wherein three physical sciences teachers and 40 learners took part in the study. The study conducted at schools of Vhuronga 2 circuit in Vhembe West District between January 2022 to November 2022. Interviews and classroom observations were used for data collections. Researchers analyzed their data through Data Analysis Scheme (DAS) which comprised of themes, categories, and characteristics in this qualitative study. Texts that belong to a particular theme were highlighted using same colour and track changes was also used to codify categories and characteristics of a theme.

**Results:** The research findings had shown numerous challenges in the teaching and learning of physical sciences including teachers and learners not used to physical sciences being taught and learnt through TSR, not familiar with some scientific words in TSR, difficulties in understanding scientific term in TSR as well as absence of Tshivenda physical sciences resources beside TSR. Consequently, this has impacts on teacher and learner’s ability to implement TSR in the teaching and learning of Physical Sciences. Moreover, the findings also show that teachers and learners participated in the study sometimes switch from Tshivenda Scientific words to English Scientific words during Physical Sciences lessons.

**Conclusions:** Therefore, it is suggested that the above-mentioned challenges in the development and application of TSR for Physical Sciences teaching need to be addressed so that teachers can teach learners Physical Sciences through language they know best. Hence, physical sciences teachers must be developed, trained, and furnished with essential language skills for them to develop Tshivenda scientific language registers on other sciences topics.

## Introduction

Before the birth of democracy in South Africa in 1994, only English and Afrikaans language were used as medium of instruction in the teaching and learning at schools. This means that African languages such as Tshivenda was not recognised within the education system. However, the beginning of democracy has brought many changes within the government organisations in South Africa. There are 11 languages which are granted official status in South Africa; two were official languages during the apartheid era (English and Afrikaans) and nine were African indigenous languages (Tshivenda, isiNdebele, Xitsonga, Sesotho, isiZulu, siSwati, Setswana, Sepedi, and isiXhosa). The constitution of the Republic of South Africa (1996) and the current language in education policy (LiEP) support what is stipulated in the South African School Act that public schools are given the opportunity to elect any of the official languages as a medium of instruction.

Some of the learners in South Africa are currently receiving their primary education through their mother tongue as a medium of instruction in the foundation phase, i.e., until Grade 3. However, starting from Grade 4 to grade 12, only English or Afrikaans is used as the language of instruction to teach and learn all of the curriculum subjects excluding the home language subject. Therefore, it is a reality in South Africa that a majority of learners are receiving their primary, secondary, and tertiary education with a language which is different to their mother tongue. A study conducted by Netshivhumbe (2018) reported that some learners who are taught through an English medium of instruction have difficulties in learning the subject’s concepts. Consequently, this is an indication that the medium of instruction which is different to the learner’s language has an impact on learners’educational achievements in a subject like Physical Sciences.

It is a reality that learners are presently receiving their school education after foundation phase through English medium of instruction regardless they are not proficient in it. Consequently, such concern gave researchers some interests of developing the Tshivenda Scientific Register (TSR) and requested that some secondary school physical sciences teachers within the Vhembe West District use it during their Physical sciences classroom practices. For this point of view the researchers investigated challenges in the teaching and learning of grade 10 physical sciences classes with the use of the Tshivenda Scientific Register (TSR).

### Literature review

Yule (2020) defines register as a conventional way of using language that is suitable in specific context, which can be identified as situational, occupational, or topical. Kabellow, Omulando, and Barasa (2019) reported that learners might employ certain registers within their learning environment which are unique to them to exclude their teachers from hearing and understanding what they are saying. Kabellow *et al.* (2019) maintain that some teachers mostly use formal English that can be understood by all the learners in the class and only sometimes employ informal English as an alternative when explaining certain concepts to the learners for them to understand what is being taught. In this study, the researchers refer to a language register as a language that is developed by academics for it to be employed in the teaching and learning of a specific subject. Furthermore, the researchers developed the Tshivenda scientific register (TSR) which needed to be employed by both teachers and learners during the entire physical sciences lessons in their classroom setting. The researchers developed the TSR to in order to examine its impact on learners’ participation and performance.

Taylor and Prinsloo (2005) reported that an English medium of instruction is a key factor delaying the progress of learners at school because these learners are required to learn and write in the medium of instruction, which is not the language they use at their households. It is important to understand that learners whose language of learning and teaching (English) differs from their home language are under extreme stress, and this has the potential to cause an underdeveloped home language ( Murphy and Evangelou, 2016). Marsh (2006) state that in other countries, attempting to learn through English has led to confusion, despair, and high drop-outs rates. Even though for the majority of learners in the Vhembe West District English is not their home language, it is still preferred as a medium of instruction which has an impact in the learning environment. In addition, Netshivhumbe and Mudau (2021) reported that leaners whose parents can’t speak English have nothing to offer to their children’s education at their household. This means that some learners only come across English at their schools and really struggle to acquire the proficiency required in the language of teaching and learning ( Madima and Makananise, 2020). Mogashoa (2017) report that it is difficult for learners to understand and conceptualise content taught when they still struggle with the language used in teaching the subject. Furthermore, Ngema (2016) observed that the problem is worsened if the science teachers are not proficient in English.

According to Botha (2022) learners who uses their mother tongue in educational settings enjoy positive learning experiences, especially if they use the language as a language of teaching and learning. Furthermore, from anecdotal evidence, learners who learn in their mother tongue have no problems with connecting newly acquired curriculum content to their existing knowledge because the processing of this new knowledge happens naturally in their mother tongue. Botha (2022) indicated that it would be a great opportunity if learners were to be educated in their mother tongue since the processing of knowledge would be easier in the mother tongue. Additionally, the use of African indigenous languages, for example, Tshivenda, as medium of instruction can increase parental support in learner’s education.

Research conducted by Awopetu (2016) reported that learners who were communicated to and instructed in their mother tongue achieved better results than their fellow participants who were communicated to and instructed in English. Botha (2022) reported that in order for children to reach their optimal potential, they need to be educated in a language that they can communicate in; a language that is comprehendible, so that they can vocally assert and express themselves. This is supported by scholar Sanchez (2013) and Chavez (2016) which indicates that learners who are taught in their mother tongue can express themselves more freely and improve their self-confidence and thinking skills. However, there are still an unavailability of physical sciences teaching and learning materials written in African indigenous languages (e.g., Tshivenda), which can be used at schools to promote mother tongue education. A study by Netshivhumbe (2018) indicated that there are teachers who do not improvise other teaching materials to assist their learners to learn the subject content as they rely on the resources provided by their schools e.g., textbook. Netshivhumbe (2018) further indicated that teachers should try to improvise teaching and learning materials where possible instead of omitting some of the activities that could possibly assist learners to develop real understanding of the subject matter.

Netshivhumbe (2018) indicated that it is a reality that some of the teachers within the Vhembe district codeswitch from English to Tshivenda to support their learners who experiences difficulties in understanding subjects’ concepts through English medium of instruction. In support of what Netshivhumbe reported, Maluleke (2019) and Sethusha (2015) indicates that some teachers during their classroom practices draw on code switching as a method of teaching to support their learners in learning and understanding the ideas of the lesson taught without difficulties. Consequently, it is a reality that some teachers use learners’ home language to facilitate the teaching and learning of physical sciences and English simultaneously. Consequently, learners are being taught bilingually.

This research sought to contribute to knowledge about the use of TSR in the teaching and learning of physical sciences and its impacts towards learners’ academic performance. The challenges regarding the change from mother tongue (Tshivenda) instruction to English instruction in South Africa has an impact on learners’ education.

### Theoretical and conceptual framework

**Figure 1.**
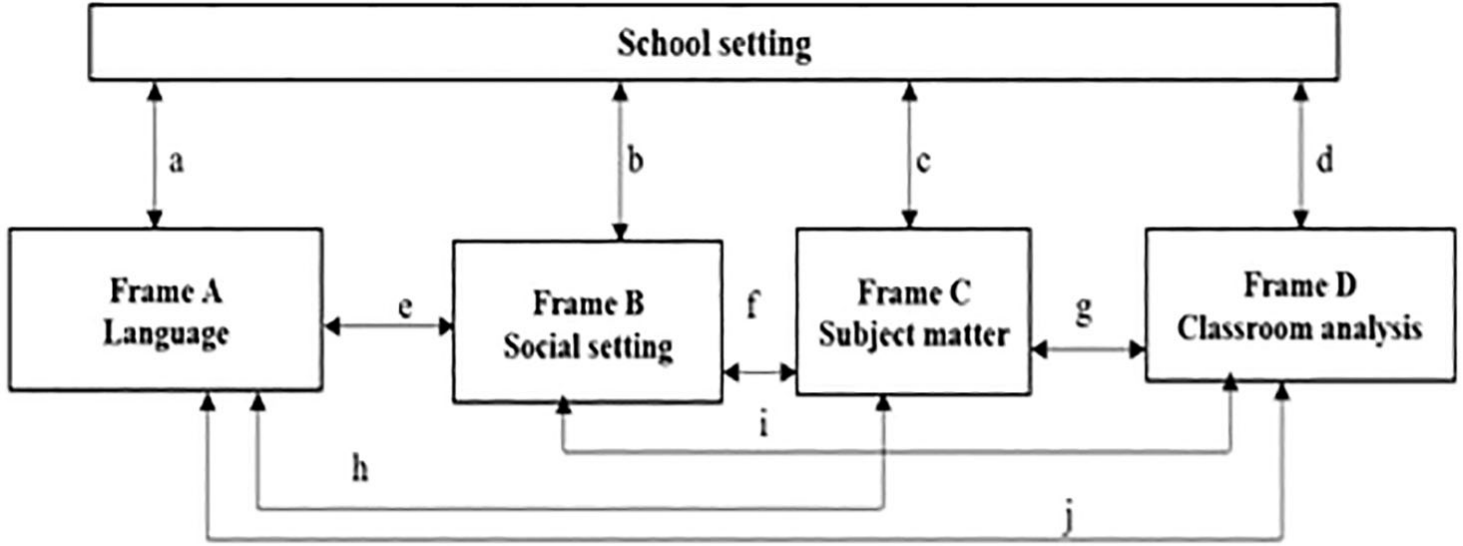
Classroom Language Investigative Framework (CLIF).

The study adopted the Classroom Language Investigative Framework (CLIF) which consists of school settings. Figure 1 illustrates the school setting as the main component that describes the school defrayals, school size, and population groups. This is where the scholars can recognise and comprehend what is truly happening in physical sciences classrooms.

CLIF assisted the researchers to diagnosed participants perceptions on developed TSR for physical sciences. Therefore, it is essential for learners to understand the language of science besides learning language of teaching and learning (LoTL). However, this can result in either subtractive or additive bilingualism. Subtractive bilingualism as defined as limitation form of bilingualism which is often connected with negative results and it applies to English second language (ESL) learners (e.g., Venda learners) as they are anticipated to become experts in English as a medium of instruction ( Lambert, 1975).

Additive bilingualism connected with a well-advanced expertise in dual languages, together with optimistic cognitive outcomes is functional in a context in which learners of any language are introduced to L2 in addition to the sustained educational use of the home language of the learner as the LoTL ( Lambert, 1975). Moreover, CLIF also assisted the researchers to know how well learners perceive scientific language on developed the TSR in the learning and teaching of physical sciences. Therefore, the researchers prepared interview tools for the purpose of being able to give responses to research questions and accomplish the objectives of this study.

### Problem of research

According to Wellington and Ireson (2008), the goal of language in science learning and teaching attracted many scholars’ interests with the principle that language is the most vital medium and a main barricade in learning science. The problem of this study is related to English as LoTL for physical sciences, basically in Vhembe West District, Limpopo Province. Vhembe West District is a mutilingual region that uses English as LoTL for curriculum subjects like physical sciences at schools. Though learners are expected to be taught and learn through English, some teachers’ and learners not proficient to teach and learn in English ( Nel and Muller, 2010). This means that there are teachers and learners are facing difficulties in their teaching and learning through the English language.

Tshotsho (2013) reports that the South African government has not delivered the human resources and physical resources required to encourage mother tongue education and English still has hegemony when compared to other indigenous languages in South Africa. This means that teachers are expected to educate physical sciences with LoTL. As a result, this paper explored some of the challenges in the application of TSR in grade 10 physical sciences. The following research questions guided the study, what are the challenges in the application of Tshivenda scientific register in the teaching and learning of physical sciences? What are the views and perceptions of physical sciences teachers and learners towards the use of the Tshivenda scientific register for physical sciences?

## Methods

### Ethical statement

This study received ethical approval from the Unisa college of education ethics review committee (2021/06/09/55131433/14/AM). Written informed consent was obtained from all participants prior to the study taking place.

### Research design

**Figure 2.**
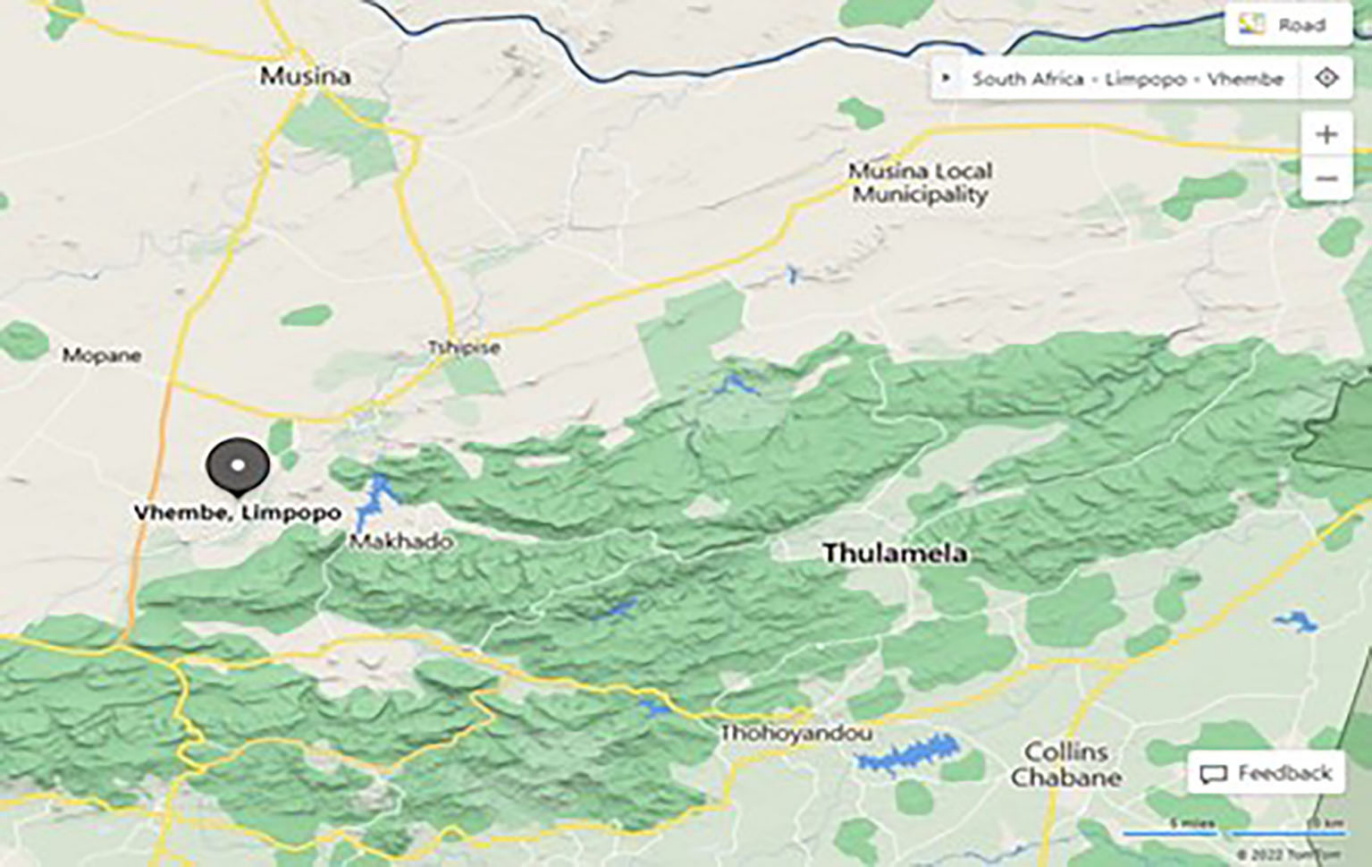
Map showing the location of the Vhembe West District.

The main purpose of this paper was to explore the impact of TSR for physical sciences teaching and learning at secondary schools. To this study, a qualitative research design was employed to provide rich descriptions of phenomena under exploration. The study targeted Physical Science teachers and learners from the Vhembe West District, Limpopo province. Furthermore, learners who were under the age of 18 years were given a consent form to ask permission from their parents for them to take part in the study. This research used an interpretative qualitative case study to develop a full understanding on the challenges in the use of developed TSR for grade 10 physical sciences. The research sites for this study were rural schools under the Makhado Local Municipality in the Vhembe West District in Limpopo province. These sites were chosen for the study as they were public schools that offer FET Phase physical sciences. The locality of Vhembe West District can be seen on Figure 2.

### Sample and participants

The sample of the study consisted of three physical sciences teachers and 40 learners from Vhembe West District, Limpopo province. In this study, purposeful sampling was used because it enables the researchers not to spend more time gathering data from participants. In short, the researchers were favoured in terms of time. In this qualitative study, purposive sampling was employed when making selections of participants for this study. This type of sampling was suitable for this study as McMillan and Schumacher (2001) reported purposive sample as the superlative selection of evidence-rich cases for an in-depth study using participants who are well-informed about the phenomenon under exploration. It was not possible for the researchers to get the entire Vhembe West District physical sciences teachers and learners to participate in the study owing to population size. Hence, purposive sampling was the most suitable sampling which ensured the researchers that only appropriate participants take part in the study.

By using a purposeful sampling, the researchers managed to include total of 43 participants according to relevant criteria, i.e., Venda teachers, and learners in FET phase physical sciences at Vhembe West District schools. This means that this study targeted participants who were currently teaching physical Sciences and competent in Tshivenda, as well as physical sciences learners in the FET phase, as they were thought by the researchers to be information-rich sources that offered valuable understandings in answering the research questions of this study. For the possibility of the study, a purposive sampling of three Physical Sciences teachers in each of the three selected secondary schools and one class of physical sciences learners from each selected school participated in the study. The researchers elected this number of participants to assure that the data collected was controllable. Purposive sampling in qualitative research includes identifying and selecting participants that have experiences and knowledge about phenomena of interest ( Annan *et al.,* 2019).

The participants selection done based on the following criteria namely, participants (teachers) must be teaching physical Sciences in FET phase schools, particularly in the Vhuronga 2 circuit; participants (both teachers and learners) must be competent in Tshivenda and participants eager to participate in the entire study. The researchers visited sampled schools and presented approval letter obtained from UNISA, LDE and circuit manager to school principals. The researchers explained the study purpose and gave school principals a letter asking permission to conduct research in their schools as well as explaining the details of the research. After obtaining permission from the schools’ principals, the letters requesting permission to conduct the study and outlining the purpose of the study were given to teachers. Learners who were under 18 were given consent form to ask permission from their parents. Thereafter, the researcher worked with the participants throughout the research process. The names of participants and schools appeared in the paper are pseudonyms and that was done to protect the identity of the participants. Hence, the selected participants assisted the researchers in answering research questions and achieving the study aim. Therefore, it was not necessary to collect data from all people in Vhembe west District to acquire valid findings.

### Data collection tools

Classroom observations and interview tools were used to gather data from physical sciences teachers and learners ( Netshivhumbe, 2023b, 2023c). Each one of us (researchers) design data gathering instruments i.e., an interview and observation tool, thereafter we had a formal meeting wherein each of us presented our data collection tools. Thereafter we had a discussion on what we should include on the proposed data collection tools. Therefore, the tools were refined and tested with one teacher and 10 learners of one school which cannot be mentioned for confidentiality purpose. The pilot study was done between 17 January to 19 January 2022 and piloting was conducted after school since it was the only time available. Additionally, the school used for piloting did not participate in this study.

February 2022 the researchers commence with the collection of data of the main study which lasted until November 2022. The researchers observed physical sciences classroom practices of teachers elected to take part in this study and all classroom observations were video- recorded by the researchers. The same applies to interviews with the participants, the researchers’ audio-taped all interviews and the language used during interviews were Tshivenda ( Netshivhumbe, 2023b). Data captured by means of recording devices were transcribed to word documents by the researchers and all translation made was done by the researchers. Physical sciences teachers and learners from three schools were interviewed in their school’s settings. Classroom observation was used in order to examine the use of TSR by both teachers and learners in class whereas the interview tools were used to gather data from participants on their views and perceptions toward TSR for physical sciences teaching and learning. Furthermore, interviews and classroom observations with the participants were recorded and transcribed and by so doing the researchers find it easier when analysing data for the study.

### Internal validity

Internal validity is explained as the extent at which the results of the research provide a true reflection of the situation ( Charamba, 2017). This definition emphasises the need for the researchers to employ content and sources that are accurate and consistent throughout. Researchers developed a data analysis scheme (DAS) and implemented it during the pilot study. The pilot study was conducted in one school with one physical sciences teacher and 10 learners who were not part of the study where proposed instruments, i.e. interview and observation tools were tested to ensure validity of this paper. On the day of piloting, teacher and learners were observed in their classroom setting where TSR was implemented in the teaching and learning of physical sciences and the observation tool was used by the researchers to evaluate what was actual happening during the lesson. Teacher and learners were also interviewed in their school setting, the teacher was interviewed before and after the lesson whereas the 10 learners were interviewed after the lesson. During piloting, it was revealed that some interviews questions were not well organised and there was a necessity to revise such questions.

The questions which were revised and modified are written in italics as follows, with the revised interview questions in bold:

- *What is your view towards the used of mother tongue instruction in the teaching and learning of physical sciences?*
- ***What is your perception towards the used of TSR in the teaching and learning of physical sciences**?* (Teacher interview question)
- *How do you rate your learners’ participation in the learning of physical sciences using Tshivenda instruction?*
- ***How do you rate your learners’ participation in the learning of physical sciences using TSR?*** (Teacher interview question)
- *How comfortable are you in learning physical sciences through Tshivenda instruction?*
- ***How did you feel about learning physical sciences through TSR?*** (Learner interview question).

It was, therefore, important for the researchers as the recorder and interpreter of the data to pay attention during observations and interviews with participants. Additionally, the researchers increased the study validity by focusing only on the data obtained from the participants of the study.

### Data analysis

In this study, the three schools (cases) were analysed and interpreted. The researchers transcribed video recorded observations and audio-recorded interviews of each case *verbatim* to a word document. Thereafter, researchers replayed video recorded observation and audio-recorded semi-structured interviews of each case to verify if the words transcribed corresponded with what was on the recording device. However, any grammatical errors displayed by participants were not corrected to ensure that the data gathered was presented accordingly and does not lose its meaning. The findings of the study were analysed using themes developed from reviewed literature and research questions. The researchers proposed the themes for the study after constructing the research questions and reviewed literature related to this study. Therefore, the themes confirmed during piloting were employed in the study includes, participants view and perceptions towards the developed TSR for physical sciences teaching and learning and experiences of participants in the teaching and learning of physical sciences through TSR. The themes recommended for the study connect for the researchers to be able to answer research questions and achieve the objectives of the study.

The researchers showed the participants their transcribed data for additions, remarks, or corrections before being considered as a final product. Thereafter, the researchers read the transcribed interviews data of each case with one theme in mind while coding until they are all finished. The data analysis scheme (DAS) that was used in the study was the one implemented during piloting. This means that the DAS suggested confirmed during pilot study before being implemented on this study. The texts that belong to a particular theme were highlighted using same colour and track changes was also used to codify categories and characteristics of a theme. The researchers went through the coded data to confirm the transcripts. The data coded were presented using narratives.

## Results

The results of this study obtained from interviews and classroom practices of three schools participated in the study. The study focuses were to divulge challenges in the use of Tshivenda Scientific Register (TSR) during physical sciences lessons. The researchers used codes to present data, for example: school 1/Teacher T1 = **S1/T1/**, school 2/Teacher T2 = **S2/T2,** school 3/Teacher T3 = **S3/T3,** Group 1/Learners 1/school 1 = **G1/L/S1,** Group 2/Learners/ school 2= **G2/L/S2,** Group 2/Learners/school 3= **G3/L/S3** ( Netshivhumbe, 2023d, 2023e).

### Challenges teachers experiences before using TSR

After the researchers developed TSR for teaching and learning physical sciences they requested the views and perception of teachers and learners toward the developed TSR. For this study purpose, the researchers only focus on the views and perceptions of teachers and learners that divulge challenges of TSR were considered. Hence, teachers were given TSR to use during their physical sciences lesson preparation and their physical sciences classroom practices. Due to the fact that implementation of TSR for physical sciences lessons, the teachers indicated the challenges they experienced during their lessons’ preparations. This is evident with the statements reported next:

“During my lesson preparation with TSR I had challenges of not knowing how to draw my lesson plan as I was used to do my physical sciences lesson through English register. Hence, my physical sciences lessons with the use of TSR take a lot of time compared to lesson I do in English. Some of the words presented in the register required me to do some consultation with tshivenda expect, i.e tshivenda educator. I had a discussion with tshivenda educator I work with where I was classified with some of the words, I was not familiar with” **S1/T1**

“My physical sciences lessons preparations with TSR was not easy even though tshivenda was my mother tongue. Teaching physical sciences with the register the researcher gave me will be my first time using the language used in the register on the entire physical sciences lessons. The language presented was pure unlike the language we use these present days of mixing tshivenda with other languages. The way the science words developed in TSR was awesome and it was not easy for me to prepare my lessons without consulting tshivenda dictionary and ask my colleagues who teaches tshivenda in my workstation” **S2/T2**

“The biggest challenge I had come across during my Physical Sciences lessons preparations is of the language that was used in the register. In our day to day lives, even though there are Physical Sciences words available in tshivenda, we normally use them in English and forget what they mean in tshivenda. Therefore, the problem is that the language we use every day is no longer a pure tshivenda and that resulted in me having difficulties in the preparation of those lessons using TSR. However, I reach out for help with some clarity in other words appeared in the TSR” **S3/T3**

### Challenges when using TSR

During physical sciences lessons where TSR was used to teach and learn, there were some learners that teachers had identified to be experiencing difficulties to know and understand science concepts. This is reported by the statements that follows:

“During the physical sciences lesson that I offered through TSR, I had noted that learners were mixing languages (Tshivenda and English). I sometimes reminded them that only the language (Tshivenda) used in the register should be used in the classroom. Some of the words presented in TSR learners were not familiar with even though they were written in their mother tongue” **S1/T1**

“During my physical science classroom practices where I was implementing TSR, I have seen my learners struggling to read and write some of the words appeared in the register. I think the cause could be that they were not use to physical sciences being taught by TSR” **S2/T2**

“The biggest challenge that learners’ experiences is the same challenge I as a teacher experience when I was doing lessons preparations which is of not knowing and understanding some of science words used in TSR. Many tshivenda words has disappeared to People including learners. Tshivenḓa language has disappeared or lost in learners as they no longer know many words in tshivenda. Ndi ngazwo nangwe maipfi a santsi kha register o nwaliwa nga Tshivenḓa, vhana vhovha vha si khou divha uri elo ipfi ndi lifhio That is why even though science words in the TSR were written in tshivenda, children were no longer knowing which word is it. You will find that a child knows that word in English but not knowing the word in tshivenda. In English it is his or her everyday language but in Tshivenḓa it seem to be new words, for example there is no child that doesn’t know fridge, each and every learner knew fridge and he or she know that if he or she put water inside the fridge, the water will change and become ice but when you talk about tshixwatudzi (fridge) a child no longer know what tshixwatudzi (fridge) is, but he or she sees tshixwatudzi (fridge) every day. When you talk about muxwatu (ice) a child doesn’t know what muxwatu (ice) is even though ice he or she sees it every day. That the challenge I saw learners experiencing when I was teaching them. Tshivenḓa language has disappear to children, some of the words they no longer know them” **S3/T3**

### Challenges physical sciences learners’ experiences in learning physical sciences when TSR was used during the lessons

The implementation of TSR in the teaching and learning of physical sciences was new to the learners. Hence, learners had challenges since they were used to physical sciences being taught and learned through the English language register. Learners expressed themselves as follows:

“We had a problem of failing to understand other words used in TSR such as mutsidi (steam), muxwatu (ice) and tshixwatudzi (fridge)” **G1/L/S1**

“Some of the words used by teachers found in TSR we did not understand words like Tshiomate, muxwatu na tshixwatudzi” **G2/L/S1**

“There are some words in the TSR which our teacher used to teach us such as tshiomate (solid), this is one of the words that we found to be difficult” **G3/L/S1**

“Some of the words used in the TSR we are not used to them in tshivenda, words like steam, ice and fridge” **G1/L/S1**

“There were words that were used in the TSR that were new to us and we failed to understand them, words like fridge, i mutsidi (steam), muxwatu (ice) and tshixwatudzi (fridge) Tshiomate (solid)” **G2/L/S2**

“Some of the words in TSR were not easy to understand because we never heard about them before, for example fridge” **G3/L/S1**

“Some of the words in TSR the teacher was talking about I was not knowing them, like the word steam” **G3/L/S2**

The use of TSR in physical teaching has resulted in teachers and learners experiencing some difficulties in the learning and teaching of physical sciences since they were used to English language register in the learning and teaching of physical science. Additionally, they were not familiar with some of the Tshivenḓa scientific words.

## Discussion

The study conducted by Botha (2022) reported that for children to reach their optimal potential, they need to be educated in a language that they can communicate in; a language that is comprehendible, so that they can vocally assert and express themselves. The above statement is supported by the study findings which revealed that teachers and learners participated in the study make use of African indigenous language i.e., Tshivenda which was the language used on the developed scientific register for physical sciences teaching and learning. Teachers (i.e., S1/T1, S2/T2, and S1/T3) used TSR during their lessons’ preparations and in their classroom practices of physical sciences. Even though TSR was implemented for teaching and learning of physical sciences, there were some challenges teachers experience in their lessons preparations as it was their first-time doing lessons preparations using TSR instead of English language register (ELR). This is an indication that teaching, and learning happened with only English instruction at school. There were few words that were presented in the developed TSR that were new to them and they used the English-Venda dictionary as well as other translation documents to understand some of the words used in TSR. Additionally, they reached out to their colleagues (Tshivenda educators) in their school setting. During classroom practices, the researchers noted that some learners did experience some difficulties in understanding some of science concepts as they were used to physical sciences concepts being written in ELR. Hence, teachers assisted those learners as they were able to explain those words that seem to be difficult for learners to understand them.

Netshivhumbe (2018) indicated that teachers should try to improvise teaching and learning materials where possible instead of omitting some of the activities that could possibly assist learners to develop real understanding of the subject matter. In support of the above statement, the study findings revealed that beside S1/T1 and S3/T3 being provided with TSR guide they make use of other teaching aids to support their learners. S3/T3 brought some resources that assisted in the teaching and learning of physical sciences, which includes stones, water, jug, beaker, containers of different shapes and other materials that were available in the classroom that relate to the lessons taught for learners to be able to understand what he was teaching using TSR. However, S1/T1 make use of objects available in the classroom which relate to the lesson taught for illustration purposes. S2/T2 physical sciences lessons were taught with the TSR as he did not improvise other teaching materials to support his learners to learn visualisation, however he gave some examples during the lessons. This is an indication that there is availability of teachers who do not improvise other teaching materials to support their teaching. This is evident in a study conducted by Netshivhumbe (2018) where it was reported that there are some teachers who do not improvise other teaching materials to assist their learners to learn the subject content as they rely only on the resources provided by their schools e.g., textbooks. Moreover, the way that physical sciences teachers (S1/T1, S2/T2, and S3/T3) taught phases of matter topic did enable the learners to take notes during the lessons since they wrote notes for learners on the chalkboard.

During physical sciences lessons, the researchers noticed that teachers assisted their learners to learn the ideas of the lesson by means of doing many explanations, using examples, doing some demonstrations, and questioning in the classroom. All the activities that S1/T1 and S3/T3 gave their learners were marked with the learners in the classroom. However, S2/T2 marked group activity by himself and no corrections was done with learners. It was also noted that there were few challenges that learners’ experiences during the application of TSR in physical sciences lessons. Mogashoa (2017) report that it is difficult for learners to understand and conceptualise content taught when they still struggle with the language used in teaching the subject. Another finding highlighted by the study is that there were words that some learners found them difficult to understand their meaning through African indigenous language used in the developed register e.g., Tshiomate (solid), muxwatu (ice), tshixwatudzi (fridge), mutsidi (steam), etc. However, the teacher was able to identify the words learners experiences some difficulties and they assisted them by explaining the words e.g., learners did not know what fridge and ice is in their mother tongue. Hence, after some clarity the teacher made on the word’s learners find them difficult in TSR, learners realised that they knew those words in English as they are used to them in the English language.

This study was limited to three selected secondary schools of Vhuronga 2 circuit in the Vhembe West District of Limpopo Province, South Africa. Only physical sciences teachers and learners of selected schools participated. For the fact that the research only focused on only three secondary schools of Vhuronga 2 Circuit in Vhembe West District may be regarded as limitation of the research. Nevertheless, through explanation offered in data analysis, the outcomes may be applicable to other districts with alike contexts.

## Conclusion

The purpose of this study was to examine some challenges teachers and learners’ experiences in the developed of TSR for physical science teaching and learning in the FET phase. The application of TSR during teacher classroom practices was not easy as Tshivenda is an African indigenous language which is still in the process of developing. Hence, Tshivenda has limited scientific terms. Most of the scientific terms presented in the TSR were translated and borrowed from English and Afrikaans. The teacher had difficulties in understanding some words which appeared in the TSR, but the teachers understood the words after reaching out to their colleagues and using other translation documents for explanations. Some of the learners indicated that they experienced challenges of failing to understand physical sciences through TSR because English is not their home language. However, few learners specified that language used in TSR is problematic because during teacher classroom practices they experienced difficulties in understanding some of the words such as tshiomate. Consequently, the teachers assisted their learners with understanding the words which were difficult to them.

The findings of this study provide evidence that there is a multiplicity of challenges in the use of TSR for physical sciences. Therefore, researchers suggested that teamwork is required and it should comprise the following people, senior citizens, physical sciences teachers, physical sciences learners, physical sciences curriculum advisors and PanSALB to develop sufficient terms for this language (Tshivenda) to be developed and not only be recognised as an official language but also as a language of teaching and learning at schools and in institutions offering higher education. Additionally, availability of literature books and multilingual natural sciences and technology term list does promise that eventually Tshivenda will be well developed like Afrikaans and English. Moreover, this study has some recommendations which need to be considered to improve the use of African languages like Tshivenda in any of the education sectors, namely, the expansion of Tshivenda scientific terminology and Tshivenda science learning and teaching materials must be prioritised; physical sciences teachers must be developed, trained and furnished with essential language skills for them to develop Tshivenda scientific language registers on other science topics; and lastly, effort should be made in developing Tshivenda as indigenous language in such a way that not only will it be recognised as official language but language of teaching and learning curriculum subjects such as physical sciences.

## Data Availability

Figshare: Teachers and learners interviews responses in Tshivenda and English, https://doi.org/10.6084/m9.figshare.22828424.v1 ( Netshivhumbe, 2023d). The project contains the following underlying data:
• S1 T1 interview responses.pdf. (Anonymised interview responses for teacher 1 in school 1 in Tshivenda with English translation). • S2 T2 interview responses.pdf. (Anonymised interview responses for teacher 2 in school 1 in Tshivenda with English translation). • School 1 group 1 learners.pdf. (Anonymised interview responses for group 1 learners in school 1 in Tshivenda with English translation). • School 1 group 2 learners.pdf. (Anonymised interview responses for group 2 learners in school 1 in Tshivenda with English translation). • School 1 group 3 learners.pdf. (Anonymised interview responses for group 3 learners in school 1 in Tshivenda with English translation). • School 2 group 1 learners.pdf. (Anonymised interview responses for group 1 learners in school 2 in Tshivenda with English translation). • School 2 group 2 learners.pdf. (Anonymised interview responses for group 2 learners in school 2 in Tshivenda with English translation). • School 2 group 3 learners.pdf. (Anonymised interview responses for group 3 learners in school 2 in Tshivenda with English translation). • School 3 group 1 learners.pdf. (Anonymised interview responses for group 1 learners in school 3 in Tshivenda with English translation). • School 3 group 2 learners.pdf. (Anonymised interview responses for group 2 learners in school 3 in Tshivenda with English translation). • School 3 group 3 learners.pdf. (Anonymised interview responses for group 3 learners in school 3 in Tshivenda with English translation). Data are available under the terms of the Creative Commons Zero “No rights reserved” data waiver (CC0 1.0 Public domain dedication). Figshare: Transcriptions of classroom observations in English and Tshivenda, https://doi.org/10.6084/m9.figshare.22828205.v1 ( Netshivhumbe, 2023e). The project contains the following underlying data:
• English lessons S3 T3.pdf (Anonymised classroom observation results for school 3 teacher in English). • English lessons S1 T1.pdf (Anonymised classroom observation results for school 1 teacher in Tshivenda and English). • English lessons S2 T2.pdf (Anonymised classroom observation results for school 2 teacher in Tshivenda and English). • Tshivenda lessons S1 T1.pdf (Anonymised classroom observation results for school 1 teacher in Tshivenda). • Tshivenda lessons S2 T2.pdf (Anonymised classroom observation results for school 2 teacher in Tshivenda). • Tshivenda lessons S3 T3.pdf (Anonymised classroom observation results for school 2 teacher in Tshivenda). Data are available under the terms of the Creative Commons Zero “No rights reserved” data waiver (CC0 1.0 Public domain dedication). Figshare: Interview tools.pdf. https://doi.org/10.6084/m9.figshare.22580209.v1. ( Netshivhumbe, 2023a). This project contains the following extended data:
• Teacher interview tool.pdf. (Blank Tshivenda questions with English translation for interviews with teachers). • Learner interview tool.pdf. (Blank Tshivenda questions with English translation for interviews with learners). Figshare: Classroom observation tool.pdf. https://doi.org/10.6084/m9.figshare.22580215.v1 ( Netshivhumbe, 2023b). This project contains the following extended data:
• Classroom observation tool.pdf (Blank copy of the interview tool used in the study to focus on teacher-learners classroom interaction and discourse). Figshare. Completed SRQR checklist for ‘Challenges in the application of the Tshivenda scientific register for Physical Sciences Classroom’. https://doi.org/10.6084/m9.figshare.22580254.v1 ( Netshivhumbe, 2023c). Data are available under the terms of the Creative Commons Zero “No rights reserved” data waiver (CC0 1.0 Public domain dedication).
