# Supplementary material for: Challenges in the application of the Tshivenda scientific register in the teaching and learning of state of matter and the kinetic molecular theory in grade 10 physical sciences classrooms
Source: F1000Res. 2023 Dec 11;12:688. Originally published 2023 Jun 16. [Version 2] doi: 10.12688/f1000research.131616.2 (PMC10904935; doi:10.12688/f1000research.131616.2)
Supplement: Peer review history

# Reviewer report: Reviewer response for version 2

**DOI:** 10.5256/f1000research.159798.r229095

**Published:** 2024-02-14

**Authors:** Tebogo Nkanyani

**Affiliations:** 1 North-West University, Mmabatho, South Africa

Review for:

**Challenges in the application of the Tshivenda scientific register for physical sciences classrooms**

 Thank you for addressing the recommendations. I have enjoyed reading the revised manuscript and have no further comments to make.

Is the work clearly and accurately presented and does it cite the current literature?

Yes

If applicable, is the statistical analysis and its interpretation appropriate?

Not applicable

Are all the source data underlying the results available to ensure full reproducibility?

Yes

Is the study design appropriate and is the work technically sound?

Yes

Are the conclusions drawn adequately supported by the results?

Yes

Are sufficient details of methods and analysis provided to allow replication by others?

Partly

Reviewer Expertise:

I am a focusing on teachind science through technological/e-learning methods. I am currently working on blended learning for teaching science, use of open education resources, use of Learning management systems for teaching science among others.

I confirm that I have read this submission and believe that I have an appropriate level of expertise to confirm that it is of an acceptable scientific standard.

# Reviewer report: Reviewer response for version 1

**DOI:** 10.5256/f1000research.144473.r221983

**Published:** 2023-11-30

**Authors:** Tebogo Nkanyani

**Affiliations:** 1 North-West University, Mmabatho, South Africa

Review for:

**Challenges in the application of the Tshivenda scientific register for physical sciences classrooms**

 This study makes a valuable contribution to the field of teaching with indigenous languages. The literature outlined elaborated well how the issue of being taught with the language apart from the mother has an impact on the learning process. However, I recommend that you address the following:

1. Title: Since the Tshivenda scientific register was designed specifically for a certain topic of Physical Sciences in Grade 10, I would recommend that you add the grade together with the topic on your title to eliminate confusion that the register covers all the Physical Sciences topics.
2. Abstract: Since you specified the type of observation used, you should also specify the type(s) of interviews used.
3. Theoretical framework: what does L2 stand for? Also indicate who authored the CLIF. Where was it adapted from? Check previous articles on how that is done.
4. Problem of research: I recommend that you put a double colon (:) before the research questions.
5. Sample and participants: You used purposeful statement, but your ideas in justifying that are not flowing, rather going back and forth. For example, the idea in line 5 of the first paragraph is the same as that of the last sentence of the second paragraph. I recommend a revision of the whole section.
6. Internal validity: I recommend that you use a table to indicate old questions and piloted (revised) questions from the pilot study outcomes.
7. Data analysis: in the first line of the second paragraph, you indicated “The researchers showed the participants their transcribed data for additions, remarks, or corrections before being considered as a final product.” Please add the rationale for doing that.
8. Discussions: line 11 – Can you be specific to the reader if you are referring to the Tshivenda speaking educators or Tshivenda language educators.

Your manuscript has several language errors. I recommend that it be language edited by a professional language editor. Also write acronyms in full, with the acronym in brackets for the first time before you repeat them as acronyms, the same way you did with the TSR acronym throughout the manuscript.

Is the work clearly and accurately presented and does it cite the current literature?

Yes

If applicable, is the statistical analysis and its interpretation appropriate?

Not applicable

Are all the source data underlying the results available to ensure full reproducibility?

Yes

Is the study design appropriate and is the work technically sound?

Yes

Are the conclusions drawn adequately supported by the results?

Yes

Are sufficient details of methods and analysis provided to allow replication by others?

Partly

Reviewer Expertise:

I am a focusing on teachind science through technological/e-learning methods. I am currently working on blended learning for teaching science, use of open education resources, use of Learning management systems for teaching science among others.

I confirm that I have read this submission and believe that I have an appropriate level of expertise to confirm that it is of an acceptable scientific standard, however I have significant reservations, as outlined above.

# Response

**Published:** 2023-12-05

**Authors:** Awelani Mudau

**Affiliations:** Science and Technology Educati, University of South Africa, Pretoria, Gauteng, South Africa

Title

 Add the grade together with the topic on your title to eliminate confusion that the register covers all the Physical Sciences topics.

 The grade and the topic on our title added.

 Abstract

 You should also specify the type(s) of interviews used.

 The type(s) of interviews used indicated and corrected as ‘Semi-structured interviews’

 Theoretical framework

 Write acronyms in full, with the acronym in brackets for the first time.

 what does L2 stand for?

 Indicate who authored the CLIF

 Acronyms written in full, with the acronym in brackets for the first time, e.g., Second Language (L2)

 L2 stand for Second Language

 The author of CLIF indicated

 Problem of research

 Put a double colon (:) before the research questions.

 Double colon (:) was used before the research questions.

 Sample and participants

 I recommend a revision of the whole section.

 Revision done on sample and participants’ section.

 Internal validity

 use a table to indicate old questions and piloted (revised) questions from the pilot study outcomes.

 Table used to present old questions and piloted (revised) questions from the pilot study outcomes.

 Data analysis

 In the first line of the second paragraph, you indicated “The researchers showed the participants their transcribed data for additions, remarks, or corrections before being considered as a final product.” Please add the rationale for doing that.

 Rationale for researchers to present participants their transcribed data for additions, remarks, or corrections before being considered as a final product included in the second paragraph in the second line to third line.

 Discussions

 Can you be specific to the reader if you are referring to the Tshivenda speaking educators or Tshivenda language educators.

 Corrected as Tshivenda speaking educators and Tshivenda language educators.

# Reviewer report: Reviewer response for version 1

**DOI:** 10.5256/f1000research.144473.r221967

**Published:** 2023-11-24

**Authors:** Sam Ramaila

**Affiliations:** 1 University of Johannesburg, Johannesburg, South Africa

This article investigated the difficulties linked to implementing the Tshivenda Scientific Register (TSR) in the classroom activities of physical sciences teachers in public secondary schools within the Vhembe West District, South Africa. Tshivenda is a language spoken in South Africa. The article discusses challenges related to incorporating the Tshivenda scientific register into physical sciences classrooms, and sheds light on the importance of considering local languages and cultural nuances in education. This is particularly relevant for promoting inclusivity and effective communication in diverse classrooms. The challenges discussed in the article touch upon issues related to teaching methodologies, instructional materials, or the adaptation of scientific concepts to the local language. Understanding these challenges can be crucial for teachers, curriculum developers, and policymakers seeking to improve science education in linguistically diverse settings. The article contribute to the broader field of research on the intersection of language and science education. Insights into challenges faced in specific linguistic and cultural contexts can inform future research and practices aimed at enhancing science education globally.

 The study provides valuable insights into the challenges faced by teachers and learners in the adoption of TSR for teaching physical sciences in Tshivenda. The challenges identified highlight the importance of considering language dynamics and familiarity in educational materials to ensure effective learning outcomes. The findings suggest a need for additional support, training, and resources to facilitate the transition to using TSR in physical sciences instruction. The study reveals significant challenges in the implementation of TSR, both in terms of teacher preparation and learner comprehension. Addressing these challenges may require targeted interventions, professional development for teachers, and modifications to the TSR to align with the language proficiency and understanding of the learners.

**1. Clarity of Purpose:**

 The paper clearly outlines its objective: to investigate challenges in the application of the Tshivenda Scientific Register (TSR) during classroom practices among physical sciences teachers in public secondary schools in the Vhembe West District, South Africa.

 The research question is well-defined, focusing on both teachers and learners' experiences with TSR in physical sciences instruction.

**2. Research Design and Methods:**The use of an interpretative qualitative case study is appropriate for exploring the challenges in-depth, involving three physical sciences teachers and 40 learners. The study duration, location, and methods (interviews and classroom observations) are clearly described, providing a comprehensive understanding of the research context.

**3. Data Analysis:**The Data Analysis Scheme (DAS) is explained, incorporating themes, categories, and characteristics. The approach aligns with qualitative research standards, allowing for a nuanced exploration of challenges in the teaching and learning of physical sciences with TSR.

**4. Results:**The presentation of results is clear and directly addresses the identified challenges, including teachers and learners not accustomed to TSR, unfamiliarity with scientific words, difficulties in understanding terms, and the absence of Tshivenda physical sciences resources. The mention of participants switching between Tshivenda and English scientific words adds depth to the findings.

**5. Conclusion:**The paper draws meaningful conclusions from the findings, emphasizing the need to address challenges in TSR development and application. The suggestion to provide training for teachers and enhance language skills to develop Tshivenda scientific language registers is a practical recommendation based on the identified challenges.

**6. Recommended minor changes to be effected**It is crucial to interpret the significant findings within the context of the employed theoretical and conceptual frameworks. Additionally, emphasizing the study's implications for science teaching and learning is essential. Moreover, it is advantageous to offer a thoughtful assessment of the reliability of the qualitative data gathered.

**7. Overall Evaluation:**The paper is well-structured, presenting a clear background, robust research design, and insightful results. The study provides valuable insights into the challenges of implementing TSR in the teaching and learning of physical sciences, emphasizing the importance of language proficiency and training for teachers. The recommendations are practical and have the potential to contribute positively to the improvement of physical sciences education in the given context. In conclusion, the paper effectively contributes to the understanding of challenges in using TSR in the context of physical sciences education and provides actionable recommendations for addressing these challenges.

**Decision: Approved**

Is the work clearly and accurately presented and does it cite the current literature?

Yes

If applicable, is the statistical analysis and its interpretation appropriate?

Not applicable

Are all the source data underlying the results available to ensure full reproducibility?

Yes

Is the study design appropriate and is the work technically sound?

Yes

Are the conclusions drawn adequately supported by the results?

Yes

Are sufficient details of methods and analysis provided to allow replication by others?

Yes

Reviewer Expertise:

Nature of science

I confirm that I have read this submission and believe that I have an appropriate level of expertise to confirm that it is of an acceptable scientific standard.
